# Durvalumab Plus High-Dose Radiation in NSCLC III: Long-Term Results from the Austrian Radio-Oncological Lung Cancer Study Association Registry (ALLSTAR)

**DOI:** 10.3390/cancers18193173

**Published:** 2026-10-01

**Authors:** Katharina Hörmandinger, Elvis Ruznic, Marisa Klebermass, Brane Grambozov, Petra Feurstein, Josef Karner, Georg Gruber, Martin Heilmann, Falk Röder, Ute Ganswindt, Danijela Minasch, Franz Zehentmayr

**Affiliations:** 1 Comprehensive Cancer Centre, Department of Radiation Oncology, Medical University Innsbruck, A-6020 Innsbruck, Austriaute.ganswindt@tirol-kliniken.at (U.G.); danijela.minasch@tirol-kliniken.at (D.M.); 2 Department of Radiation Oncology, Paracelsus Medical University, A-5020 Salzburg, Austria; e.ruznic@salk.at (E.R.); j.karner@salk.at (J.K.); f.roeder@salk.at (F.R.); 3 Klinikum Ottakring, A-1160 Vienna, Austria; marisa.klebermass@gesundheitsverbund.at (M.K.); doktor@petra-feurstein.at (P.F.); 4 Ordensklinikum Linz, A-4010 Linz, Austria; 5 Comprehensive Cancer Centre, Department of Radiation Oncology, Medical University Vienna, A-1090 Vienna, Austria; martin.heilmann@akhwien.at

**Keywords:** Durvalumab, NSCLC, real world data, chemoradiotherapy, radiation dose escalation, intrathoracic tumour control

## Abstract

**Simple Summary:**

Locally advanced non-small-cell lung cancer (NSCLC) is a main cause of cancer morbidity and mortality worldwide. Surgical options are limited in higher stages. In recent years, more and more studies have shown better outcomes when combining immunotherapies with the standard of care (SoC) chemoradiotherapy (CRT) treatment in unresectable NSCLC UICC stage III. ALLSTAR is a nationwide prospective registry study to collect and analyze data of 228 patients on their outcomes in a long-term follow-up. In accordance with the so-called PACIFIC regimen, a higher OS, PFS, TTDM, and TDLR was demonstrated when adding Durvalumab after CRT. Results also showed a promising association between radiation dose escalation combined with Durvalumab and higher intrathoracic tumour control.

**Abstract:**

Purpose: Chemoradioimmunotherapy (CRIT) with 60–66 Gy is the standard of care (SoC) for non-small-cell lung cancer (NSCLC) UICC stage III. The aim of this real-world data (RWD) study was to report the long-term impact of Durvalumab and radiation dose escalation on clinical outcomes. Material and Methods: ALLSTAR is a nationwide prospective multicentre registry for NSCLC UICC stage III, in which 12/14 Austrian centres participated (ethics approval number: 1002/2019). The basis for the current analysis is the third data extraction, which was completed on 2 June 2025. Apart from the SoC, alternative dose-fractionation and sequential CRT concepts >66 Gy were also permitted. Durvalumab maintenance treatment for one year was administered at the discretion of the tumour board at each centre. Results: Compared to prior analyses by our group, 40 individuals who completed follow-up (FUP) were added to this final analysis, leading to a total patient population of 228 (156 CRT plus Durvalumab; 72 CRT). In 156 patients, Durvalumab was added to CRT, while 72 received CRT only. The median FUP periods were 41.0 (95%-CI: 36.7–45.4) and 37.8 months (95%-CI: 31.4–44.1) for the Durvalumab and CRT cohort, respectively. Radiation dose escalation combined with Durvalumab treatment was associated with a nominally higher ITC of 37 months (95%-CI: 24.5-not reached) compared with 17.7 months (95%-CI: 14.9-not reached) in patients receiving neither dose escalation nor Durvalumab (*N* = 135; HR = 0.61; 95%-CI: 0.37–1.006; *p*-value 0.051). With Durvalumab maintenance, a median PFS of 22.8 months (95%-CI: 19.4–38.3) was observed compared to 16.5 months (95%-CI: 12.6–24.5) without (*N* = 228; HR = 0.61; 95%-CI: 0.42–0.88; *p*-value 0.0071). Median OS was 52.6 months (95%-CI: 39.5-not reached) with Durvalumab compared to 27.4 months (95%-CI: 18.7-not reached) without (*N* = 228; HR = 0.52; 95%-CI: 0.34–0.78; log-rank *p*-value < 0.002). Median time to death or distant metastasis (TTDM) was 33.3 months (95%-CI: 23.5–45) in the Durvalumab cohort compared to 18.7 months (95%-CI: 14.8-not reached) in the CRT cohort (*N* = 228; HR = 0.60; 95%-CI: 0.42–0.89; log-rank *p*-value 0.01). Median time to death or local relapse (TDLR) with Durvalumab was 30.4 months (95%-CI: 23.5–51.1) compared to 17.2 months (95%-CI: 14.8–25.7) without (*N* = 226; HR = 0.58; 95%-CI: 0.40–0.83; log-rank *p*-value < 0.003). Conclusion: This final RWD analysis implies both a beneficial effect of high-dose irradiation combined with Durvalumab on ITC and the long-lasting impact of Durvalumab on PFS, OS, TTDM, and TDLR.

## 1. Introduction

Non-small cell lung cancer (NSCLC) accounts for approximately 80% of all lung cancers [1]. One third of these patients present in locally advanced stage UICC IIIa-c [1], which in fact comprises a quite heterogeneous patient population.

The prospective randomized trials conducted between the mid-1990s and 2015 [2,3,4,5,6,7] have established concomitant CRT (cCRT) with total radiation doses of 60–66 Gy as the SoC in the pre-immunotherapy era. Nevertheless, the sequential mode (sCRT) is still an option for frail elderly patients who are not deemed fit enough for concomitant treatment. Furthermore, the PACIFIC trial with CRT followed by 1-year Durvalumab maintenance yielded 33.1% PFS and 42.9% OS at five years [8,9,10]. These results—achieved at well-manageable toxicity rates—mark a breakthrough in the field of NSCLC research. In the wake of the introduction of Durvalumab in thoracic oncology, PACIFIC-R was launched to validate the real-world effect of this regimen in daily clinical practice [11,12,13,14]. With 1399 patients from 11 countries worldwide, it is the largest longitudinally collected dataset in the field. Apart from this comprehensive initiative, many other smaller and mostly single-institutional analyses [15,16,17,18,19,20,21,22,23,24] corroborate the results from PACIFIC and PACIFIC-R. Although the Austrian radio-oncological lung cancer study association registry (ALLSTAR) also aligns with PACIFIC-R, two conceptual differences stand out. Firstly, the focus was placed on the integration of immunocheckpoint inhibition with radiation dose escalation. Secondly, because the initial idea of the registry was to get an overview of the treatment landscape for this disease entity in Austria, about one third of the patients in ALLSTAR had CRT only, which serves as a comparator for the Durvalumab group. In this respect, ALLSTAR resembles more the original PACIFIC trial [8,9,10] than other RWD studies.

A previous analysis based on the second database read-out demonstrated 2-year rates of 56% and 71% for PFS and OS with ICI, respectively, which were significantly better than without. At 2 years, the local control (LC) rate was 80% compared to 65% with high-dose radiation >66 Gy, which was slightly more pronounced (82%) when Durvalumab was added [25,26]. Other RWD studies report improved LC with Durvalumab [17,18,21], which is why we hypothesized that a combined effect of Durvalumab plus dose escalation might result in clinically relevant differences in intrathoracic disease control (ITC). In the current analysis of the third and final database read-out after four planned follow-up visits, we report long-term PFS, OS, and TTDM as well as exploratory endpoints such as ITC and TDLR.

## 2. Methods

### 2.1. Study Design and Patients

ALLSTAR was designed as a prospective multicentre registry study with the aim of collecting nationwide data from patients diagnosed with NSCLC UICC stage III (Approved by the ethics committee of the Federal State of Salzburg 2020/03/20 under the following number: 1002/2019). While real-world data (RWD) on this topic have been published before [10,15,16], the distinctive features of our study are its prospective nature and the inclusion of patients not enrolled in early access programs (EAP). In this context, we want to emphasize that ALLSTAR—although prospective—is not a randomized control trial. With the PACIFIC trial [8,9,10] in mind, we aimed to gain deeper insight into the treatment regimens used across Austrian radiation oncology departments, with regard to radiation doses and fractionation as well as systemic treatment regimens, particularly the use of Durvalumab as maintenance therapy, and their impact on oncological outcomes and toxicity. In contrast to prior publications by our group, which also included patients who—in the wake of neoadjuvant approaches—received immune-checkpoint inhibitor (ICI) therapy before radiotherapy (RT), the current analysis compares patients who received the PACIFIC regimen compared with those who had CRT without immunotherapy. Another difference is the fact that all patients recruited until the end of the recruitment period, i.e., 2023/09, have now finished the preplanned four follow-up visits. Therefore, this final enlarged cohort consists of 228 patients, which is 40 patients more compared to previous analyses [25,26]. Inclusion criteria comprised an interdisciplinary tumour board decision recommending CRT for unresectable UICC stage III NSCLC (TNM version 8) in patients aged 18 or older with histologically or cytologically proven NSCLC. For detecting intrathoracic relapses, the following diagnostic procedures were applied. If—based on a thoracic contrast-enhanced CT scan, which was used as the standard follow-up imaging—lesions suspicious for recurrences in the thorax were found, PET-CT was performed. Subsequently, pathology verification using EBUS/TBNA and bronchoscopy or transthoracic biopsy was pursued. Finally, the staging was completed by cranial MRI or contrast-enhanced CT. Further details on diagnostic work-up and follow-up were published elsewhere [25,26].

### 2.2. Radiochemoimmunotherapy

Although treatment decisions were up to the participating radiation oncology centres, the minimum requirement for RT was conventional 3D planning. Total radiation doses of 60–66 Gy with 2 Gy per fraction were considered as SoC, but alternative dose-fractionation concepts were also permitted. Hence—for reasons of comparability—radiation doses were converted into biologically equivalent dose in 2 Gy fractions (EQD2) according to the following formula:

EQD2 = D*(d + α/β)/(2 + α/β)

with D as total dose, d as single fraction, and an α/β-value of 10 assumed for tumour tissue. Administering chemotherapy as a platinum-doublet concurrently to radiation is usually considered the SoC [2,3,4,5,6,7], although sCRT was also possible. The addition of Durvalumab maintenance treatment after completing CRT was at the discretion of the involved tumour board. Some centres—at least initially—followed the treatment approach published by the PACIFIC working party in 2017 [8], but finally the much-criticized European Medicines Agency (EMA) decision [27] was adopted.

### 2.3. Endpoints and Statistics

Primary endpoints for this final outcome analysis were PFS, OS, and—assessing the combined effect of Durvalumab and applied radiation dose–ITC, which was defined as absence of progression in all of the following sites: primary tumour, regional lymph nodes (either irradiated or unirradiated), pleura, and contralateral lung. In accordance with PACIFIC-R [13], we also included exploratory endpoints such as TTDM and—again with a focus on localized intrathoracic treatment effect –TDLR. Finally, treatment-related toxicity was graded according to CTCAE v5.0 with a focus on esophageal and pulmonary side effects as adverse events of special interest (AESI). The current paper is based on the third data extraction, which was completed on 2 June 2025, so that the minimum follow-up for censored patients was two years as of histopathological verification. Patients lost to follow-up were censored for OS at the date of the last recorded contact with the treating physician. The Mann–Whitney test was used for subgroup comparisons of patient and treatment characteristics as well as side effects. The following variables entered the multivariate models: age, gender, ECOG, smoking status, UICC stage, tumour histology, gross tumour volume GTV_Tumour_, programmed death ligand 1 (PD-L1) status, concurrent or sequential CRT, and Durvalumab treatment.

The statistical software R (v 4.5.1) [28] was utilized, along with the “tidyverse” [29] and “ggplot2” [30] packages for data curation and plotting. Survival analyses including Kaplan–Meier, Cox proportional hazards modelling, and restricted mean survival (RMST) estimations were performed using the *survival* [31], *survminer* [32], and *survRM2* [33] packages. To compare subgroups, log-rank tests were conducted with the day of histopathological diagnosis as index date. The Benjamini–Hochberg (BH) [34] correction was applied to adjust for multiple testing. Since the Cox model assumes that hazard ratios for any of the covariates remain constant over time [35], we tested the proportional hazards assumption using Schoenfeld residuals from the R survival [31] package. *p*-values below 0.05 were considered statistically significant.

## 3. Results

### 3.1. Patients

After closing recruitment in 2023/09, the current analysis complements previously published data [25,26] from the Austrian radio-oncological study association registry (ALLSTAR) with respect to clinical outcome and toxicity. Of the 188 patients in these prior publications, 17 were excluded since they received immunotherapy other than Durvalumab before the start of RT in the wake of upcoming neoadjuvant chemoimmunotherapy trials for NSCLC UICC stage III patients [36,37,38]. In addition to these 171 patients, 57 prospectively collected patients were included in the current analysis, leading to a total patient population of 228. Slightly more than two-thirds of them (156/228) received Durvalumab after cCRT or sCRT (Table 1). By the time of database readout (2 June 2025), 109/228 (47.8%) of patients were still alive, 97/228 (42.5%) had died, and 22/228 (9.6%) were lost to follow-up. A total of 156 patients (68.4%) received Durvalumab following CRT, whereas 72 (31.6%) did not receive immunotherapy. Median age was 67.4 years (range 36.4–84.5) in the Durvalumab group and 66.3 years (range: 44.2–90.7) in the control group. About 60% were male, and most patients had a good general condition with ECOG 0–1, although patients with a higher PS were also included. PD-L1 status was known in >90% with a cutoff of 1%. Similar to all other baseline characteristics, UICC stages were equally distributed between groups (see Table 1).

### 3.2. Chemoradioimmunotherapy

The majority of patients received sCRT, with 109/156 (68.4%) in the Durvalumab cohort and 42/72 (71.2%) in the CRT cohort. The median number of chemotherapy cycles was 2 (range: 1–11) and 3.5 (0–7) in the Durvalumab and the control arm, respectively (*p* = 0.554, Table 2). Depending on histology, platinum (cisplatinum or carboplatinum) was combined with one of the following substances: taxane, pemetrexed, vinorelbine, gemcitabine or etoposide. One patient had 11 cycles of chemotherapy, i.e., three cycles of cisplatinum and vinorelbine before RT followed by treatment de-escalation to vinorelbine only, due to reduced kidney function. In 12/228 cases (5.3%), chemotherapy was omitted, mostly due to a reduced ECOG performance status (5 out of 12 were classified as ECOG 2 before treatment initiation). Consistent with the original PACIFIC publication [8], 10.3% of Durvalumab-treated patients had a PD-L1 expression of less than 1%, whereas 33.3% of patients without immunotherapy had an expression greater than 1%. In 15 patients, the PD-L1 status was unknown. Twenty-four PD-L1-positive patients did not receive Durvalumab for one of the following reasons: pulmonary toxicity after CRT, autoimmune disorders, reduced general condition, and death shortly after completing CRT. The median time interval between initiation of Durvalumab and the end of CRT was 14 days (range: −20 to 380). Of note, one patient received the first dose of Durvalumab before completing RT. With 94.4%, the majority of patients started ICI treatment within 42 days after completion of CRT. The median number of immunotherapy cycles was 13, with a wide range of 1–38 (Table 2). Forty of the 156 patients (25.8%) received 21 or more cycles of Durvalumab. As for radiation therapy (Table 2), the most frequently used treatment technique was intensity-modulated RT/volumetric arc therapy (IMRT/VMAT), applied in >97% of patients. In exceptional cases (<3%), patients were treated with conventional 3D conformal RT. GTV_Tumour_ was significantly (*p* = 0.003) smaller in the Durvalumab group (median: 44.4 mL, range 0.0–483.8) compared with the CRT only group (median: 67.0, range 1.0–784.1), resulting in a significantly (*p* < 0.001) higher EQD2 at the primary tumour site for these patients (median: 66 Gy, range: 0.0–100.0) than for the CRT only group (median: 60 Gy, range 24.8–96.0). The median administered dose to the involved lymph nodes was identical in both groups (EQD2 = 57.3 Gy), although with wide ranges in both groups (31.3–70.0 Gy versus 24.0–81.3 Gy; *p*-value = 0.010; Table 2).

### 3.3. Clinical Outcomes

The overall median follow-up time was 39.6 months (95%-CI: 35.3–43.8), with 41.0 (95%-CI: 36.7–45.4) versus 37.8 months (95%-CI: 31.4–44.1) for the Durvalumab and CRT-only cohorts, respectively.

#### 3.3.1. Intrathoracic Tumour Control

**Figure 1:**
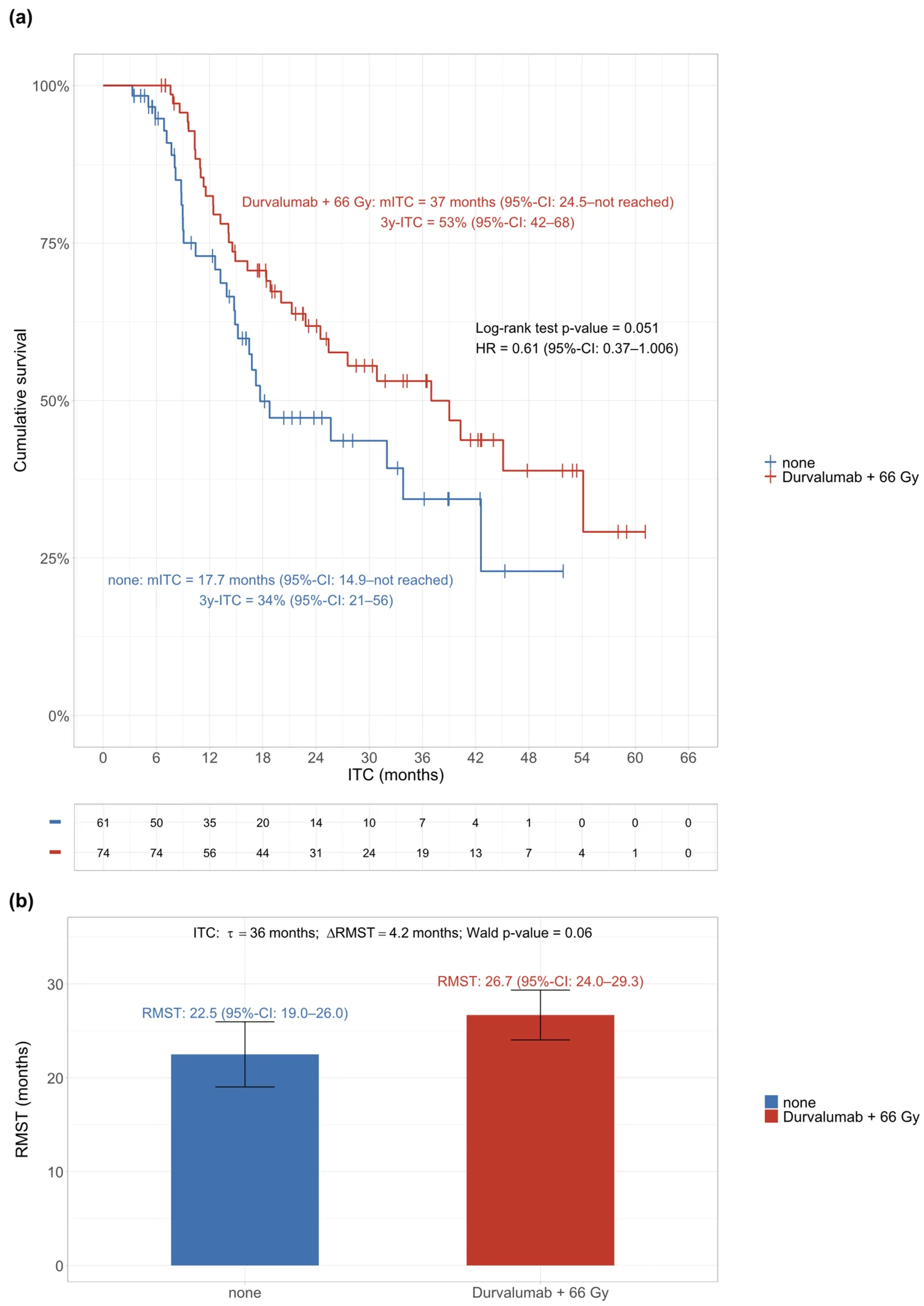
Intrathoracic tumour control (ITC). (**a**) Patients who received Durvalumab treatment combined with radiation dose escalation of 66 Gy or more (*n* = 74; 34 events) had a nominally better ITC compared to those patients who had neither of the two modalities (*n* = 61; 29 events). This difference reached borderline statistical significance (*N* = 135; HR = 0.61; 95%-CI: 0.37–1.006; log-rank *p*-value 0.051; Schoenfeld residuals test *p*-value 0.32). (**b**) The restricted mean survival time (RMST) analysis at 36 months revealed a difference in favour of combined treatment (Durvalumab + 66 Gy) of 4.2 months (Wald test *p*-value 0.06).

Ninety of the 156 (57.7%) patients treated in the Durvalumab arm experienced intrathoracic relapse, compared with 39/72 (54.2%) in the non-Durvalumab group. Quantitatively, the sites of intrathoracic relapse did not differ significantly between the two therapy regimens (Table 3). Overall, most relapses occurred in the contralateral lung or pleura (54/228 events; 23.7%), followed by simultaneous recurrence in the primary tumour and the involved lymph nodes (39/228 events; 17.1%) as well as isolated relapses in the tumour itself (25/228 events; 11.0%). Exclusive lymph node failure occurred in 4.8% of all patients (Table 3). Radiation dose escalation (total doses >66 Gy EQD2) combined with Durvalumab treatment was associated with an approximately 40% lower risk of intrathoracic relapse compared with patients receiving neither dose escalation nor Durvalumab (*N* = 135; HR = 0.61; 95%-CI: 0.37–1.006; *p*-value 0.051; Figure 1a). Median ITC was 37 months (95%-CI: 24.5-not reached) in the combined treatment group compared to 17.7 months (95%-CI: 14.9-not reached) in the group that received neither of the two treatment modalities. The 3-year ITC for the first group and the second was 53% (95%-CI: 42–68) and 34% (95%-CI: 21–56), respectively. RMST analysis at this time point showed an ITC-difference of 4.2 months in favour of the combined treatment (Figure 1b).

#### 3.3.2. Progression-Free Survival

**Figure 2:**
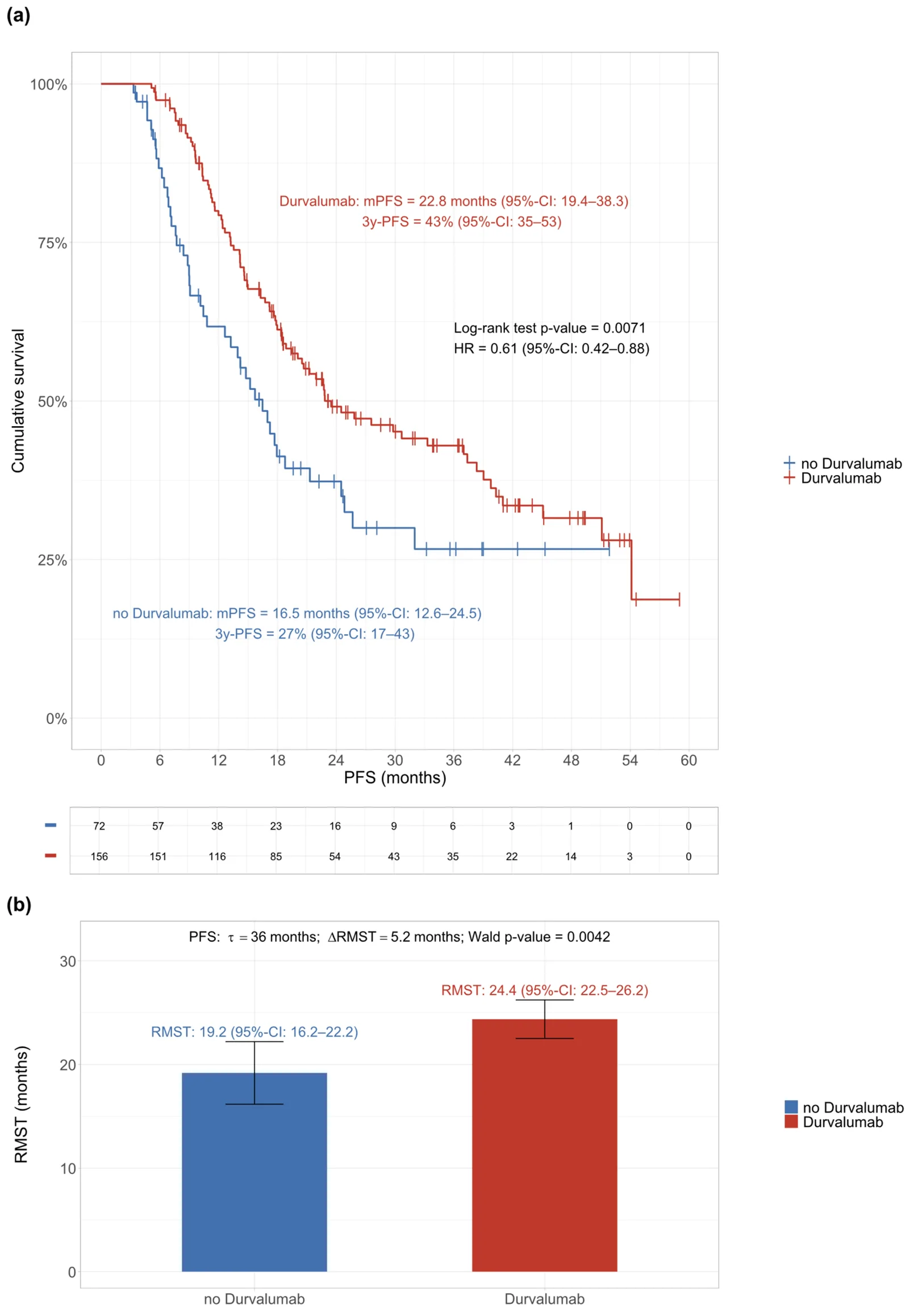
Progression-free survival (PFS). (**a**) Patients who received Durvalumab treatment (*n* = 156; 88 events) had significantly better PFS compared to those without (*n* = 72; 43 events): *N* = 228; HR = 0.61; 95%-CI: 0.42–0.88; log-rank *p*-value 0.0071 (Schoenfeld residuals test *p*-value 0.0014). (**b**) The restricted mean survival time (RMST) analysis at 36 months revealed a significant difference of 5.2 months in favour of Durvalumab (Wald-test *p*-value = 0.0042).

With 131/228 (57.5%) events, the overall median PFS was 21.3 months. As shown in Figure 2, Durvalumab treatment was associated with an improved PFS compared to CRT alone (*N* = 228; HR = 0.61; 95%-CI: 0.42–0.88; *p*-value 0.0071). While in the first group, a median PFS of 22.8 months (95%-CI: 19.4–38.3) and a 3-year PFS of 43% (95%-CI: 35–53) were observed, the latter showed a median PFS of 16.5 months (95%-CI: 12.6–24.5) and a 3-year PFS of 27% (95%-CI: 17–43). The RMST at 3 years revealed a PFS difference of 5.2 months in favour of immunotherapy (Figure 2).

#### 3.3.3. Overall Survival

**Figure 3:**
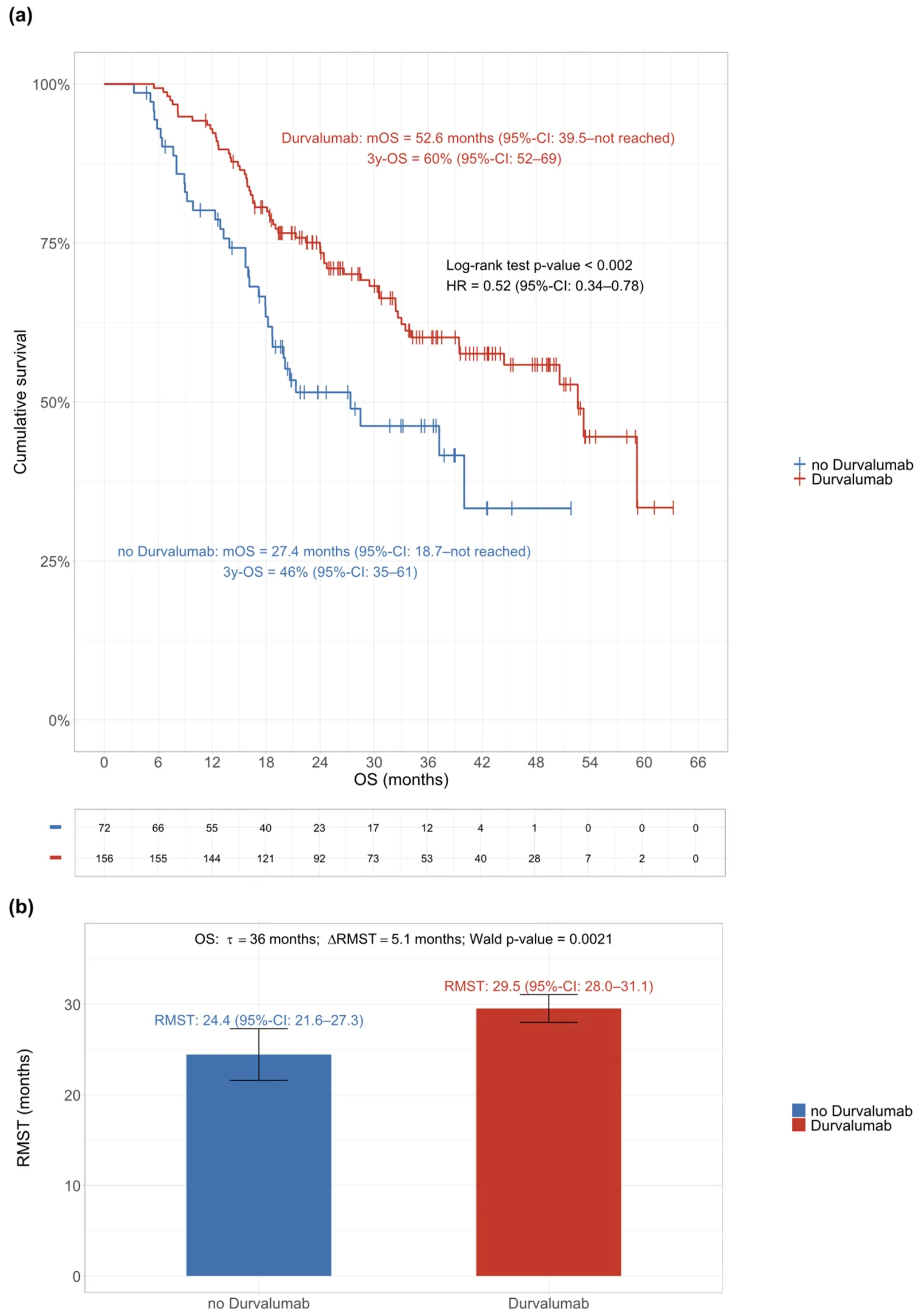
Overall survival (OS). (**a**) Patients who received Durvalumab treatment (*n* = 156; 61 events) had significantly better OS compared to those without (*n* = 72; 36 events): *N* = 228; HR = 0.52; 95%-CI: 0.34–0.78; log-rank *p*-value < 0.002 (Schoenfeld residuals test *p*-value = 0.24). (**b**) The restricted mean survival time (RMST) analysis at 36 months revealed a difference in favour of Durvalumab treatment of 5.1 months (Wald test *p*-value 0.0021).

At the time of data extraction 109/228 (57.5%) patients were still alive. While 61/156 (39.1%) deaths were observed in the Durvalumab group, 36/72 (50%) were seen in the non-Durvalumab sub-cohort. Median OS was 52.6 months (95%-CI: 39.5-not reached) compared to 27.4 months (95%-CI: 18.7-not reached), demonstrating a significant survival benefit for patients with durvalumab maintenance (*N* = 228; HR = 0.52; 95%-CI: 0.34–0.78; log-rank *p*-value < 0.002; Figure 3a). A 3-year OS rate of 60% (95%-CI: 52–69) was observed in the Durvalumab group compared to 46% (95%-CI: 35–61) in the other group (Figure 3a). Additionally, the RMST analysis at 3 years demonstrated an OS benefit of 5.1 months for patients treated with Durvalumab (Figure 3b).

#### 3.3.4. Time to Death or Distant Metastasis

**Figure 4:**
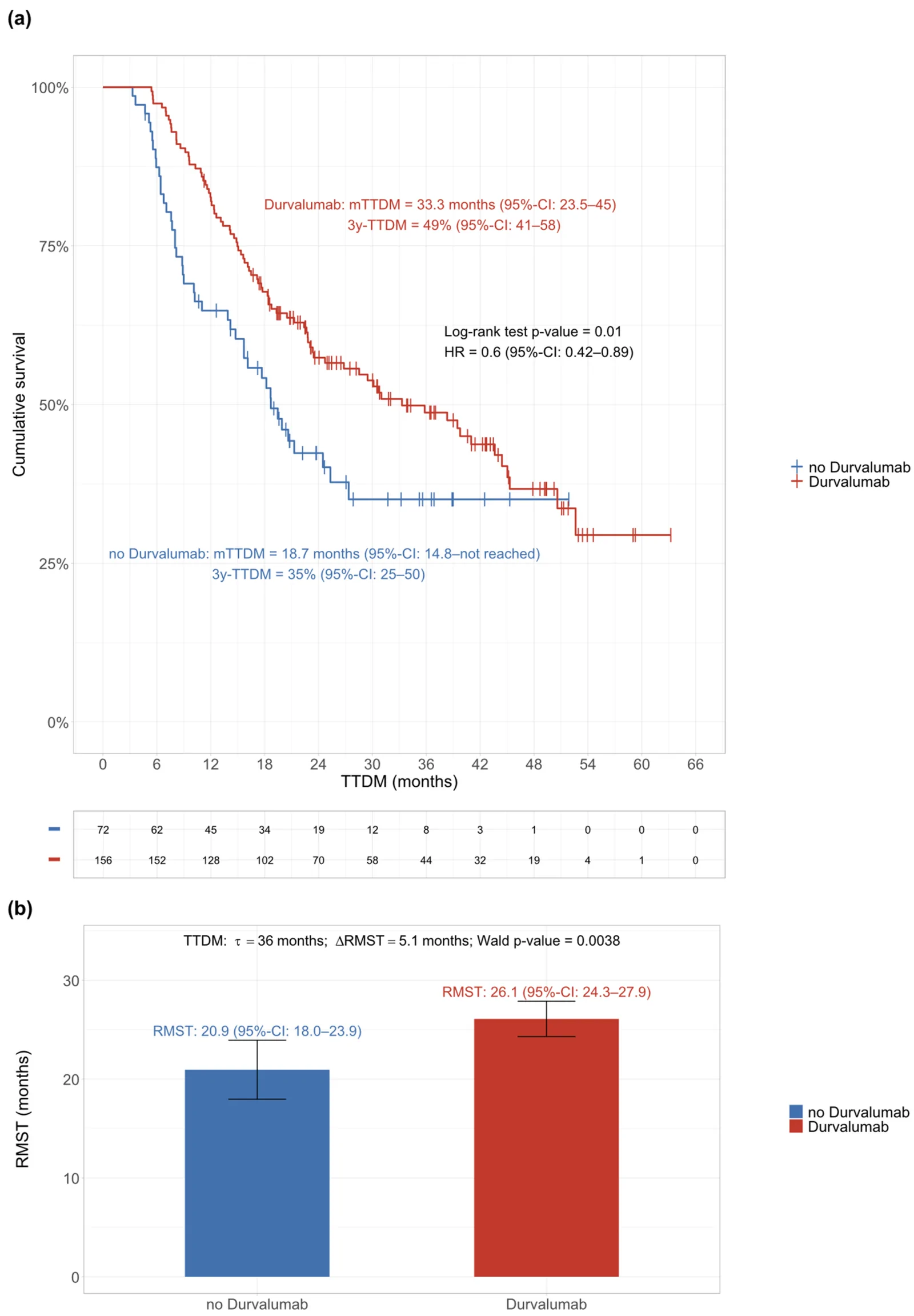
Time to death or distant metastasis (TTDM). (**a**) Patients who received Durvalumab treatment (*n* = 156; 83 events) had significantly better TTDM compared to those without (*n* = 72; 42 events): *N* = 228; HR = 0.60; 95%-CI: 0.42–0.89; log-rank *p*-value 0.01 (Schoenfeld residuals test *p*-value 0.02). (**b**) The restricted mean survival time (RMST) analysis at 36 months revealed a significant difference of 5.1 months in favour of Durvalumab treatment (Wald-test *p*-value = 0.0038).

Median TTDM was 33.3 months (95%-CI: 23.5–45) in the Durvalumab cohort, compared to 18.7 months (95%-CI: 14.8-not reached) in the non-Durvalumab group. The 3-year TTDM was 49% (95%-CI: 41–58) and 35% (95%-CI: 25–50), respectively, which marks a significant difference in favour of Durvalumab treatment (*N* = 228; HR = 0.60; 95%-CI: 0.42–0.89; log-rank *p*-value 0.01; Figure 4a). Likewise, the RMST analysis at 3 years detected a TTDM-difference of 5.1 months in favour of Durvalumab (Figure 4b).

#### 3.3.5. Time to Death or Local Relapse

**Figure 5:**
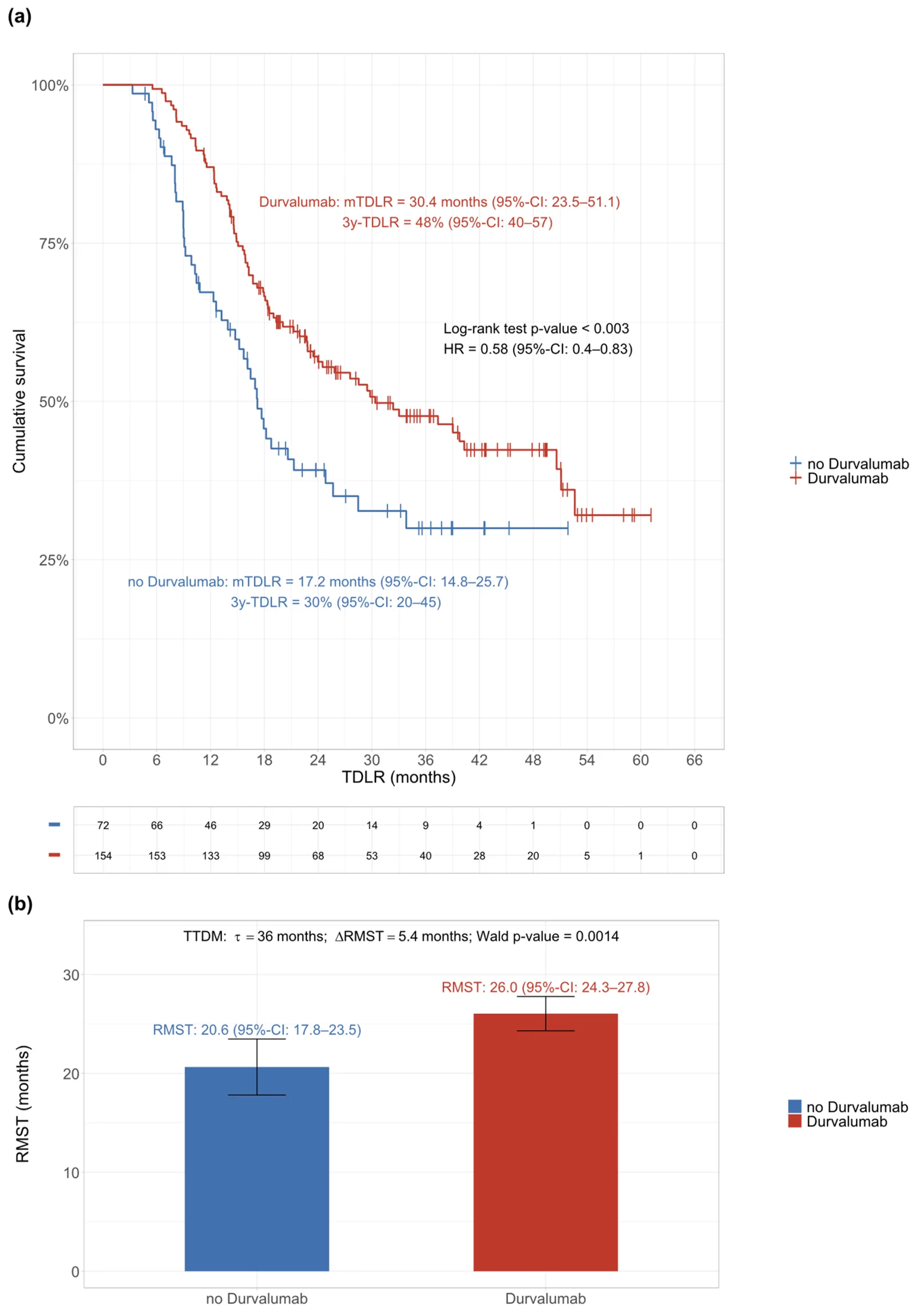
Time to death or local relapse (TDLR). (**a**) Patients who received Durvalumab treatment (*n* = 156; 81 events) had significantly better TDLR compared to those without (*n* = 72; 45 events): *N* = 226; HR = 0.58; 95%-CI: 0.40–0.83; log-rank *p*-value < 0.003 (Schoenfeld residuals test *p*-value 0.036); (**b**) The restricted mean survival time (RMST) analysis revealed a significant difference of 5.4 months in favour of Durvalumab (Wald-test *p*-value = 0.0014).

Our analysis reveals that patients who were treated with Durvalumab had a significantly longer TDLR (*N* = 226; HR = 0.58; 95%-CI: 0.40–0.83; log-rank *p*-value < 0.003) than those without. Median TDLR for the Durvalumab cohort was 30.4 months (95%-CI: 23.5–51.1) compared to 17.2 months (95%-CI: 14.8–25.7) in the non-Durvalumab group. The 3-year TDLR rates were 48% (95%-CI: 40–57) and 30% (95%-CI: 20–45), respectively (Figure 5a). Again, RMST at this time point showed a significant TDLR advantage for the Durvalumab group (Figure 5b) with 26.0 (95%-CI: 24.3–27.8) versus 20.6 months (95%-CI: 17.8–23.5).

### 3.4. Toxicity

Since esophagitis and pneumonitis are the most important side effects in thoracic RT, these toxicities were considered AESIs. A similar frequency was observed in both therapy groups for esophagitis (all grades) as shown in Table 4 (*p* = 0.855). No grade 4 or grade 5 esophagitis was documented. As for pneumonitis, the overall rate was higher in the Durvalumab group with 53/156 (34%) compared to the 15/72 (21%) cases in the patients without ICI (*p* = 0.050). Apart from pneumonitis, eighteen cases of ICI-related side effects were reported, namely seven cases of pancreatitis, four cases of thyroiditis, and singular cases of hepatitis, colitis, hypophysitis, dermatitis, and exanthema. Altogether, three pneumonitis-related deaths occurred: two in the Durvalumab and one in the non-ICI arm. In the first group, both of the patients had UICC stage IIIC squamous cell carcinoma. One of them—with pre-therapeutic COPD grade III—died of respiratory insufficiency diagnosed with pneumonitis 2.5 months after completing CRT. This patient’s treatment regimen consisted of three cycles of induction chemotherapy with carboplatinum and paclitaxel, radiation of the primary tumour (GTV size: 7 mL) with a total dose of 100 Gy EQD2 and 49 Gy EQD2 to involved lymph nodes, followed by three cycles of Durvalumab. The second patient received three cycles of cisplatinum and vinorelbine concurrently with RT, followed by four cycles of Durvalumab. The total radiation dose was 70 Gy EQD2 to the primary tumour (GTV size: 177 mL). He was diagnosed with pneumonitis 3.1 months after completion of RT. In the second group, grade 5 pneumonitis occurred 5.2 months after RT in one patient with UICC stage IIIA who suffered from pre-existing cardiac co-morbidity. The treatment regimen consisted of four cycles of chemotherapy followed by RT (GTV size: 2.2 mL; total EQD2 60 Gy). Additionally, one case of death due to bilateral pneumonia 121 days after completion of cCRT, containing four cycles of cisplatin combined with vinorelbine and a total EQD2_Tumour_ of 38 Gy. For further toxicities see Table 4.

### 3.5. Durvalumab Treatment and ECOG Impact Clinical Outcome

**Figure 6:**
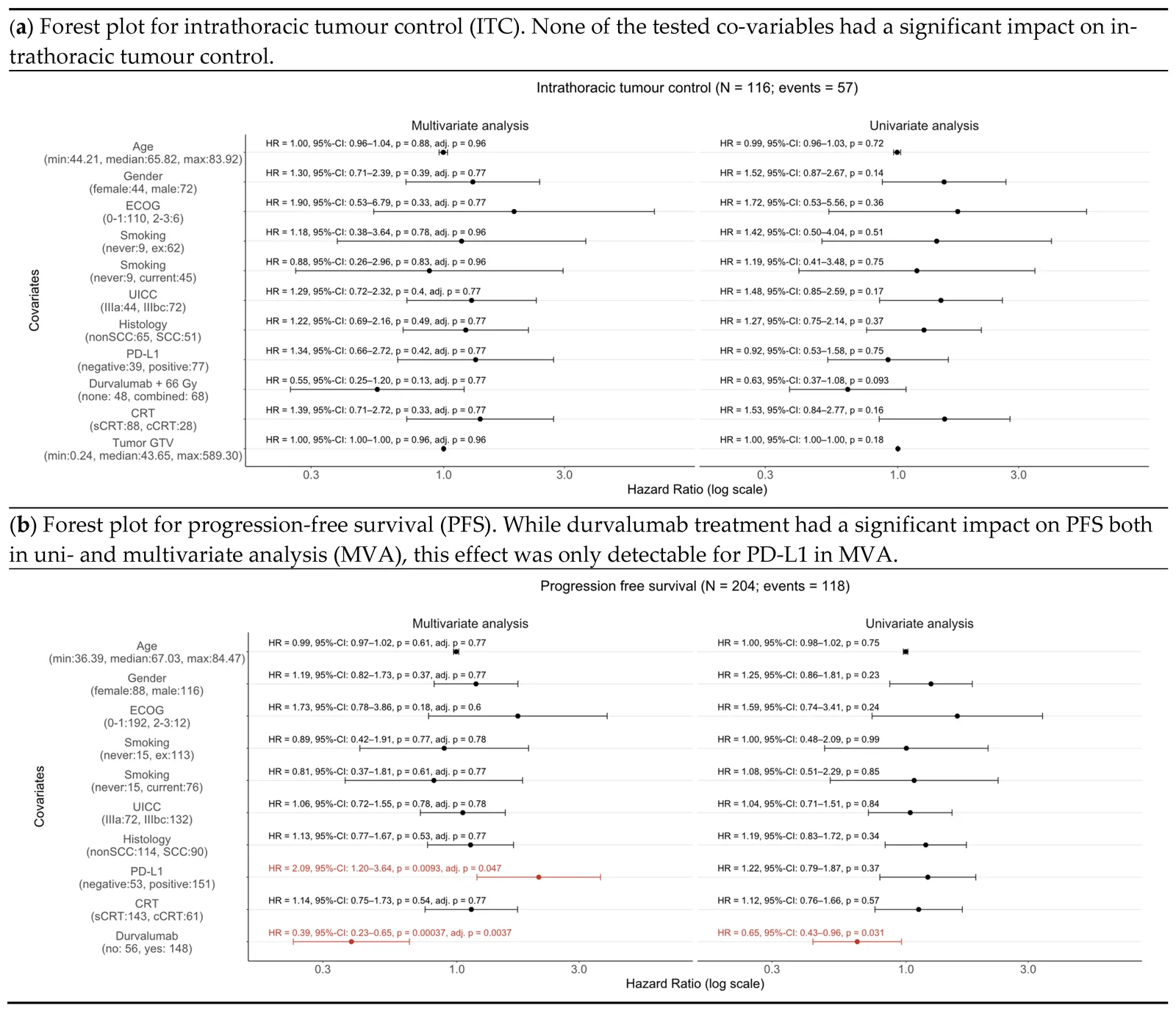

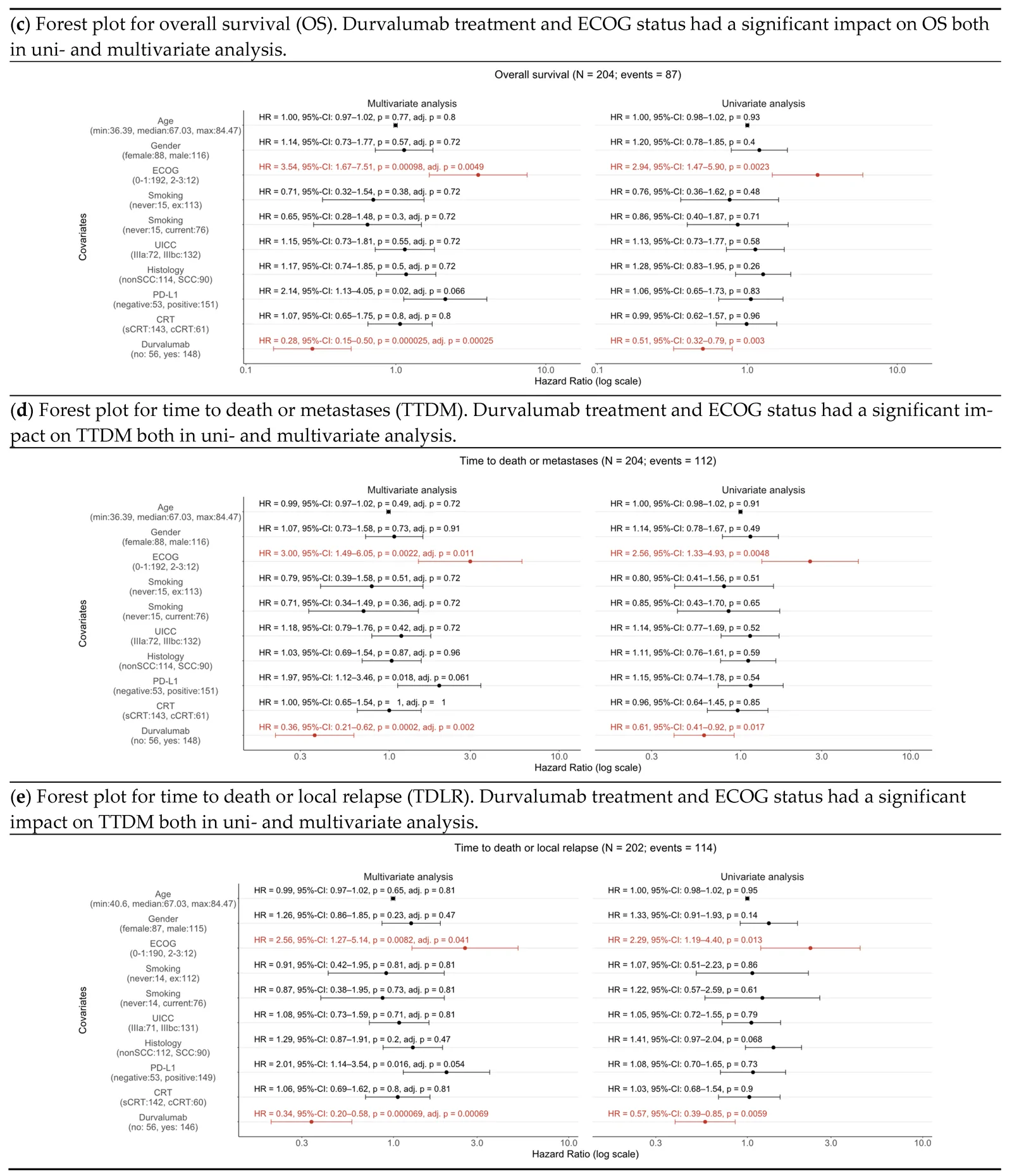
Association between patient and treatment characteristics with outcome: (**a**) intrathoracic control, (**b**) progression-free survival, (**c**) overall survival, (**d**) time to death or metastases, (**e**) time to death or local relapse.

In order to evaluate the effect of patient and treatment characteristics on outcome parameters (ITC, PFS, OS, TTDM, and TDLR), different covariates such as age, gender, ECOG, smoking status, UICC stage, tumour histology, GTV_Tumour_, PD-L1 status, concurrent versus sequential CRT, and Durvalumab treatment were tested in univariate (UVA) and multivariate analyses (MVA). Regarding ITC, no significant predictors could be detected (Figure 6a). Concerning PFS, Durvalumab treatment showed a significant impact (HR = 0.39; 95%-CI: 0.23–0.65; adj. *p* = 0.0037), whereas a borderline effect of PD-L1 status was discernable in the MVA (HR = 2.09; 95%-CI: 1.20–3.64; *p* = 0.0093; adj. *p* = 0.047; Figure 6b). When testing the previously mentioned parameters on their impact on OS (Figure 6c), Durvalumab (HR = 0.28; 95%-CI: 0.21–0.62; adj. *p* = 0.002) and ECOG (HR = 3.00; 95%-CI: 1.49–6.05; adj. *p* = 0.011; see also Supplementary Figure S1) were found to have a significant effect (Figure 6c). Likewise for TTDM, significant impacts of Durvalumab (HR = 0.36; 95%-CI: 0.21–0.62; adj. *p* = 0.002) and ECOG (HR = 3.00; 95%-CI: 1.49–6.05; adj. *p* = 0.011; Supplementary Figure S2) could be demonstrated (Figure 6d). Finally, the aforementioned two parameters, i.e., Durvalumab (HR = 0.34; 95%-CI: 0.20–0.58; adj. *p* = 0.00069) and ECOG (HR = 2.56; 95%-CI: 1.27–5.14; adj. *p* = 0.041; see also Supplementary Figure S3), were also significant covariates for TDLR (Figure 6e).

## 4. Discussion

This final analysis of the nationwide prospective ALLSTAR on CRIT for unresectable stage III NSCLC reports outcomes with a minimum follow-up of two years. We demonstrate that radiation dose escalation combined with Durvalumab consolidation might be associated with improved ITC. As expected [8,9,10], Durvalumab significantly improved clinical outcomes, i.e., OS, PFS, TTDM, and TDLR.

Multiple RWD studies have demonstrated the utility of the PACIFIC regimen in daily clinical practice [16,17,18,19,20,39,40,41,42,43,44,45]. Among these, ALLSTAR is one of the largest nationwide, prospective datasets with mature follow-up. A typical feature of RWD studies is a higher median age compared to prospective trials [10], which is also the case in ALLSTAR (67 years). The oldest patient was over 90 years at diagnosis. In line with prior publications [16,17,18,19], most patients were male and current or former smokers. Unlike PACIFIC-R [12], the ALLSTAR includes about 7% of patients with reduced performance status (ECOG 2–3), which better reflects patient populations in routine practice. Consistent with PACIFIC [8], PD-L1–negative patients were also treated with Durvalumab at the beginning of the recruitment period for ALLSTAR. Later on, these patients did not receive ICI due to EMA restrictions. This decision was criticized sharply given the biological heterogeneity of PD-L1 and practical concerns regarding biopsy-taking [27]. Notably, the rate of missing PD-L1 status in PACIFIC-R [11] was 31%, with up to 90% in some other real-world datasets [24]. Compared to these numbers, the PD-L1 testing rate in ALLSTAR was extremely high, with 94.9% in the Durvalumab and 91.3% in the CRT-only cohort, respectively.

With 37.3% of all patients, ALLSTAR includes a considerable number of patients treated with total radiation doses above 66 Gy EQD2, whereas the rate of patients treated with high-dose RT is usually below 10% in other RWD studies [11,23,45,46,47]. ALLSTAR therefore helps to fill the evidence gap on radiation dose escalation in the ICI era. With about 70%, sCRT was the most commonly used regimen in ALLSTAR. While many studies required cCRT for inclusion [16,18,46,48,49] and cCRT is associated with better OS [7,50], it is not always feasible in practice due to comorbidities and/or logistics. The inclusion of frail patients (ECOG 2–3) in ALLSTAR likely contributed to the higher proportion of sCRT. Another reason might be the fact that two high-volume centres adhere to radiation dose escalation practices, rendering the concomitant approach too toxic. Extending results from prospective trials such as PACIFIC-5 [51] and PACIFIC-6 [52], our findings support the notion that sCRT is a valid curative treatment option.

With regard to the administration of ICI, PACIFIC [8,9,10] reports better outcomes with early start of Durvalumab, i.e., within 14 days after CRT. This is in line with the median interval of 14 days in ALLSTAR, with the majority of cases (94.4%) beginning ICI treatment within 42 days. Biologically, this improved treatment response to ICI may be explained by upregulation of PD-L1 expression after RT. In vitro experiments showed that the radiogenic PD-L1 increase decreased after 7 days, indicating that early initiation of immunotherapy may be clinically relevant [53].

While RWD studies in this field usually emphasize systemic effects of immune checkpoint inhibition, radiation oncology as a localized treatment modality shifts the focus to the thorax where—at least theoretically—RT and ICI can unfold a combined effect. RT induces immunogenic cell death by releasing tumour-related antigens and danger-associated molecular patterns, which results in immigration of dendritic cells followed by T-cell activation via tumour-antigen presentation. The local activation of the immune system may trigger T-cell-mediated antitumoural responses in unirradiated areas [54], which is usually denoted as the “abscopal effect”. The current analysis is the first to report intrathoracic disease control as a combined effect of radiation dose escalation and immunotherapy. Total RT doses >66 Gy EQD2 together with Durvalumab resulted in a 40% risk reduction of intrathoracic relapse (HR 0.61; 95%-CI: 0.37–1006; Figure 1). While this result differs from RTOG 0617 [6], it is partially in line with some RWD studies that report improved LC with Durvalumab [17,18,21]. These studies report 1-year LC rates of 75–86%. In ALLSTAR, ITC was consistent with the combination of dose-escalation plus Durvalumab, which is markedly higher than in historical series [7]. Of note, this was also corroborated by RMST. On top of that, the 5-year analysis of PACIFIC [10] reports a 10% increase in long-term overall response rate (ORR) with Durvalumab and therefore concludes that Durvalumab may also improve “local disease control”. Although this term is not well defined by the authors, there seems to exist a localized, intrathoracic effect of Durvalumab, which could be potentially enhanced with higher radiation doses.

Compared to a median follow-up of 41.0 months in the ALLSTAR Durvalumab sub-cohort—although not reaching the over 60 months in the latest PACIFIC-R publication [12]—most other RWD studies [17,18,19,20,23,48,55] have shorter follow-up than ours. Our data revealed a median PFS of 22.8 months (43% at 3 years) and a median OS of 52.6 months (46% at 3 years) in the Durvalumab group, which is broadly in the range of PACIFIC-R and other RWD series [12,13,21,46,56]. Durvalumab—as expected [8,9,10]—significantly improved both PFS and OS compared to CRT alone. In order to make our study more comparable to PACIFIC-R [13], we also assessed TTDM and TDLR, which are exploratory endpoints that have demonstrated their utility in thoracic oncology [13,20,57,58]. Median TTDM was 33.3 months in our Durvalumab cohort, which is in the same range as in published RWD analyses [13,20]. This finding supports the role of immunotherapy for distant tumour control and OS. The second, above-mentioned endpoint combining local tumour control and OS, TDLR, is rarely presented. In fact, Filippi et al. were the first to report this endpoint [13]. They found a 3-year TDLR of 66%, which is 18% higher than in ALLSTAR. This discrepancy may be partly explained by poorer performance status and more advanced tumour stages with UICC IIIC in one-fifth of the total ALLSTAR patient population.

Regarding pulmonary toxicity, we found nominally higher rates in the Durvalumab versus CRT-only subgroup (Table 4). PACIFIC-R, which did not use a standardized toxicity grading system, reports 19.4% of patients with “symptomatic pneumonitis” requiring cortisone treatment [14]. This number is in the same range as the approximately 21% that we found in our Durvalumab sub-cohort (Table 4). Similarly, pneumonitis rates in other RWD studies [21,46,55] were higher after ICI than with CRT alone. This is of special concern, as pneumonitis is known to be a relevant reason for Durvalumab discontinuation [17,18,20,39,46,59], which—in turn—impacts OS. Wheatley-Price et al. reported a median OS of 41.3 months for patients with Durvalumab discontinuation after an AESI, whereas the median OS was not reached in patients having completed ICI therapy [45]. Notably, the fact that in two of the three cases of fatal pneumonitis the total EQD2 amounted to >66 Gy may be a hint that the combination of fierce dose escalation and immunotherapy has to be applied cautiously. However, because the number of events was small, we did not perform dose category-specific analysis of pulmonary toxicity. Therefore, safety conclusions regarding radiation dose escalation combined with Durvalumab remain preliminary unless prospective studies in larger datasets with predefined toxicity assessments are conducted.

Since the first publication of the PACIFIC study, almost a decade has passed, which has seen major advances in multi-omics in patient selection for NSCLC stage III. PD L1 is an important biomarker, but its predictive value depends on clinical and biological context. Moreover, it cannot be regarded as a binary switch between either sensitivity or resistance to ICI. Meta-analyses have demonstrated the clinical benefit from ICIs even in PD L1-negative patients [60,61,62]. PD-L1 expression can be heterogeneous and sample-dependent or also change over time with various treatment modalities [62]. This highlights the importance of taking into consideration the whole genetic and epigenetic picture rather than just focusing on a single marker. However, other mutations like STK11, KEAP1, and NFE2L2, together with tumour mutational burden and the tumour microenvironment, also play an important role.

Somatic alterations such as STK11/LKB1 and KEAP1 have been associated with adverse prognosis in lung adenocarcinoma and squamous cell carcinoma [63,64]. KEAP1-loss-of-function and NFE2L2-gain-of-function mutations are found in both histological subtypes. The NFE2L2 gene encodes NRF-2, which induces antioxidant effects. Under physiological conditions, NRF-2 is degraded by KEAP1. Finding these mutations more frequently in smokers might indicate their antitumoural function, while at the same time they may reduce sensitivity to therapies whose mode of action is oxidative stress, such as radiotherapy [65]. In this context, co-mutational effects may be important. Worse survival was found in patients harbouring STK11, KEAP1 and KRAS mutations at the same time [66], whereas a single STK11 mutation had no impact on tumour progression in KRAS wild-type NSCLCs [63]. The functional complexity of these co-mutational interchanges is demonstrated by the fact that STK11/KEAP1 mutated cells show a decreased PD-L1 expression, whereas KRAS mutated cells present a higher PD-L1 status and are therefore more likely to respond well to immunotherapy [67].

Tumour mutational burden (TMB) and neoantigen load are additional determinants of immunotherapy efficacy. A retrospective multi-centre analysis demonstrated that a very high TMB—defined as at least 19 mutations per megabase—was significantly associated with improved PFS and OS in chemoimmunotherapy for advanced NSCLC [61,63,68].

Last but not least, the tumour microenvironment (TME) modulates anti-tumour immunity through multiple mechanisms. Cellular signalling either works via direct cell-to-cell contact, such as PD-L1/PD-1 or CTLA4/B7 interaction, thereby decreasing immune response. Also, the major histocompatibility complex (MHC) binding to the T-cell receptor complex is a common example and works via antigen presentation to T-cells. Soluble mediators such as interleukins and interferons further shape the dynamics of T-cell recruitment. Interleukin-2, for instance, promotes proliferation of cytotoxic CD8+ T-cells on the one hand but also supports regulatory T-cells on the other, while TGF β exerts immunosuppressive effects [69]. Interferon-gamma usually boosts antitumour immunity by enhancing antigen presentation and activating cytotoxic CD8+ T-cells and natural killer cells but has an immunosuppressive effect after chronic exposure [70]. CRT can alter the TME and might therefore change treatment response over time [69]. RT generates DNA damage, which leads to enhanced TMB and—secondarily—promotes MHC expression and cytotoxic T cell priming [71]. In contrast, RT can induce TGF-β, which suppresses CD4+ T-cell differentiation and reduces CD8+ T-cells [72].

The current analysis has the following limitations. First, three high-volume centres contributed approximately 80% of the cohort, which reduces the generalizability of the results, although 12 of the 14 Austrian RT centres participated in the registry. Also, treatment decisions were centre-specific rather than protocolized, leading to interinstitutional variability. This approach systemically introduces both selection and treatment biases, which should be kept in mind, especially in the context of the uneven distribution of PD-L1 positivity. The magnitude of the Durvalumab effect with respect to the various clinical endpoints may therefore have been overestimated. In this context, the administration of Durvalumab was initially based on the original PACIFIC study itself [8,9], while in the course of data acquisition the participating centres were obliged to implement the EMA requirements more stringently [27]. It has to be acknowledged that this shift, which did not take place at the same time in every centre, may constitute a source of bias. Secondly, consistent with other real-world registries, follow-up schedules were less standardized than in randomized control trials (RCTs), potentially prolonging time-to-event estimates. In this context, the so-called immortal time bias also may artificially prolong PFS and OS in the Durvalumab group since patients had to be progression-free and alive to be eligible for ICI treatment. In the current patient population, the interval between CRT and the beginning of Durvalumab was very short (median 14 days), which may limit the impact of immortal time bias although it cannot be entirely excluded. Thirdly, when choosing ITC and TDLR as endpoints, a certain degree of subjectivity cannot be denied since there was no blinded independent centralized review (BICR) of follow-up imaging. Additionally, due to the observational design of the study, the correlation between radiation dose escalation and tumour control can be regarded as purely descriptive. Both the uneven distribution of Tumour-GTV sizes and EQD2 across the Durvalumab and non-Durvalumab groups, as well as the fact that higher radiation doses were predominantly delivered at high volume centres, may constitute a bias that should be kept in mind when interpreting the ITC improvement. Finally, COVID-19 restrictions might have had an impact on follow-up intervals, symptom reporting, and the attribution of pulmonary toxicity events. Despite these shortcomings, the statistical framework strengthens the robustness of our results with respect to the presented clinical endpoints. While single hazard ratios can be misleading when hazards are time-dependent and the proportional hazards assumption is violated, RMST compares the area under the survival curve up to a prespecified time point. A significant RMST difference, as observed here, demonstrates a clinically meaningful survival benefit within the chosen follow-up window.

## 5. Conclusions

ALLSTAR is a nationwide prospective registry on NSCLC stage III that provides longitudinal evidence on the clinical use of the PACIFIC regimen. This current analysis suggests a combined effect of high-dose irradiation and Durvalumab on ITC, thereby expanding the comparatively small evidence base in regard to local treatments. It also complements results from previous publications by our group and other RWD studies by showcasing the long-lasting impact of Durvalumab on PFS, OS, TTDM, and TDLR.

## Figures and Tables

**Table 1:** Patient characteristics (AC = adenocarcinoma, SCC = squamous cell carcinoma, NOS = not otherwise specified).

Patients *N* = 228				
		Durvalumab	No Durvalumab	*p*-Value
		*n* = 156	*n* = 72	
Sex	male	89 (57.1)	44 (61.1)	0.563
	female	67 (42.9)	28 (38.9)	
Age (years)	median	67.4	66.3	0.868
	range	36.4–84.5	44.2–90.7	
Smoking status	never	14 (9)	3 (4.2)	0.410
	ex	86 (55.1)	40 (55.6)	
	current	56 (35.9)	29 (40.3)	
ECOG	0–1	146 (93.6)	65 (90.3)	0.376
	2–3	10 (6.4)	7 (9.7)	
Histology	AC	84 (53.8)	31 (43.1)	0.124
	SCC	67 (42.9)	35 (48.6)	
	NOS	5 (3.2)	6 (8.3)	
PDL-1	<1%	16 (10.3)	41 (56.9)	<0.001
	>1%	132 (84.6)	24 (33.3)	
	unknown	8 (5.1)	7 (9.7)	
T-stage	Tis	0 (0.0)	0 (0.0)	0.293
	1	22 (14.2)	9 (12.5)	
	2	21 (13.5)	11 (15.3)	
	3	49 (31.6)	17 (23.6)	
	4	59 (38.1)	35 (48.6)	
	unknown	5 (3.2)	0 (0.0)	
N-stage	0	13 (8.3)	7 (9.7)	0.287
	1	12 (7.7)	9 (12.5)	
	2	92 (59.0)	33 (45.8)	
	3	39 (25.0)	23 (31.9)	
M-stage	0	156 (100)	72 (100)	
	1	0 (0.0)	0 (0.0)	
UICC	IIIa	56 (36.4)	28 (38.9)	0.746
	IIIb	68 (44.2)	28 (38.9)	
	IIIc	30 (19.5)	16 (22.2)	

**Table 2:** Treatment characteristics (CRT = chemoradiotherapy; VMAT = volumetric arc therapy; IMRT = intensity modulated radiotherapy; EQD2 [Gy] = biologically equivalent dose in 2 Gy fractions; GTV [mL] = gross tumour volume; 3-D = three dimensional).

Treatment *N* = 228					
			Durvalumab	No Durvalumab	*p*-Value
			*n* = 156	*n* = 72	
Treatment sequence	concomitant CRT		47 (30.1)	17 (28.8)	0.851
	sequential CRT		109 (69.6)	42 (71.2)	
RT Technique	VMAT/IMRT		152 (97.4)	70 (97.2)	0.925
	3-D		4 (2.6)	2 (2.8)	
Tumour	EQD2	median	66	60	<0.001
		range	0.0–100.0	24.8–96.0	
	GTV	median	44.4	67.0	0.003
		range	0.0–483.8	1.0–784.1	
Lymph nodes	EQD2	median	57.3	57.3	0.010
		range	31.3–70.0	24.0–81.3	
	GTV	median	29.0	30.4	0.204
		range	1.0–317.9	1.9–473.0	
Elective nodal irradiation	EQD2	median	50	54.0	0.245
		range	32.5–60.0	44.3–60.0	
	GTV	median	86.0	145.0	0.853
		range	2.0–429.0	8.8–265.0	
Chemotherapy cycles *	median		2	3.5	0.554
	range		1–11	0–7	
Durvalumab cycles	median		13	-	-
	range		1–38	-	

* platinum (cis-/carboplatinum) combined with taxane, pemetrexed, vinorelbine, gemcitabine, or etoposide.

**Table 3:** Sites of intrathoracic relapse.

Intrathoracic Relapses			
	Durvalumab	No Durvalumab	*p*-Value
	*n* = 156 (86.3%)	*n* = 72 (31.7%)	
Tumour (isolated)	18 (11.5)	7 (9.7)	*p* = 0.683
Tumour + lymph nodes	26 (16.7)	13 (18.1)	*p* = 0.796
Lymph nodes (isolated)	8 (5.1)	3 (4.2)	*p* = 0.753
Contralateral lung/Pleura	38 (24.5)	16 (22.2)	*p* = 0.706

**Table 4:** Toxicity.

Toxicity *N* = 228				
		Durvalumab	No Durvalumab	*p*-Value
		*n* = 156	*n* = 72	
Esophagitis	Grade 1	19 (12.3)	6 (8.3)	0.855
	Grade 2	50 (32.3)	24 (33.3)	
	Grade 3	4 (2.6)	2 (2.8)	
	Grade 4	0 (0.0)	0 (0.0)	
	Grade 5	0 (0.0)	0 (0.0)	
Pneumonitis	Grade 1	20 (12.8)	6 (8.3)	0.050
	Grade 2	29 (18.6)	7 (9.7)	
	Grade 3	1 (0.6)	1 (1.4)	
	Grade 4	1 (0.6)	0 (0.0)	
	Grade 5	2 (1.3)	1 (1.4)	
ICI-related	any grade	18 (11.5)	0 (0.0)	0.030
Other	any grade	17 (10.9)	9 (12.5)	0.723

## Data Availability

The original contributions presented in this study are included in the article. Further inquiries can be directed to the corresponding author.

## Supplementary Materials

The following supporting information can be downloaded at: https://www.mdpi.com/article/10.3390/cancers18193173/s1, Supplementary Figure S1: Impact of performance score (ECOG) on overall survival (OS). (a) Patients with a better performance score (ECOG 0-1; n = 211; events: 85) had significantly better OS (mOS = 50.6 months; 95%-CI: 39.4-not reached) compared to those with a higher ECOG of 2-3 (n = 17; events: 12; mOS = 18.4 months; 95%-CI: 14-not reached): HR = 3.1; 95%-CI: 1.6-5.6; log-rank *p*-value < 0.0002 (Schoenfeld residuals test *p*-value = 0.66). (b) The restricted mean survival time (RMST) analysis at 36 months revealed a difference in favour of better performance score (ECOG 0-1) of 10.2 months (Wald test *p*-value 0.00016). Supplementary Figure S2: Impact of performance score (ECOG) on time to death or metastases (TTDM). (a) Patients with a better performance score (ECOG 0-1; n = 211; events: 112) had a significantly better TTDM (mTTDM = 30.7 months; 95%-CI: 22.8-43.6) compared to those with a higher ECOG of 2-3 (n = 17; events: 13; mTTDM = 17.8 months; 95%-CI: 8.2-not reached): HR = 2.3; 95%-CI: 1.2-4.1; log-rank *p*-value < 0.004 (Schoenfeld residuals test *p*-value = 0.68). (b) The restricted mean survival time (RMST) analysis at 36 months revealed a difference in favour of better performance score (ECOG 0-1) of 8.6 months (Wald test *p*-value 0.0011). Supplementary Figure S3: Impact of performance score (ECOG) on time to death or local relapse (TDLR). (a) Patients with a better performance score (ECOG 0-1; n = 209; events: 113) had a significantly better TDLR (mTDLR = 28.5 months; 95%-CI: 22.7-39.8) compared to those with a higher ECOG of 2-3 (n = 17; events: 13; mTDLR = 15.9 months; 95%-CI: 9.0-not reached): HR = 2.5; 95%-CI: 1.4-4.4; log-rank *p*-value < 0.002 (Schoenfeld residuals test *p*-value = 0.59). (b) The restricted mean survival time (RMST) analysis at 36 months revealed a difference in favour of better performance score (ECOG 0-1) of 9.5 months (Wald test *p*-value 0.00014).

## Abbreviations

AESI	adverse event of special interest
ALLSTAR	Austrian Radio-Oncological Lung Cancer Study Association Registry
BH	Benjamini–Hochberg
BICR	blinded independent centralized review
cCRT	concomitant chemoradiotherapy
CRIT	chemoradioimmunotherapy
CRT	chemoradiotherapy
EAP	early-access programme
EMA	European Medicines Agency
EQD2	biologically equivalent dose in 2 Gy fractions
GTV	gross tumour volume
ICI	immune-checkpoint inhibitor
ITC	intrathoracic tumour control
IMRT	intensity-modulated radiotherapy
LC	local control
MHC	major histocompatibility complex
MVA	multivariate analysis
NSCLC	non-small-cell lung cancer
OS	overall survival
PD-L1	programmed death ligand 1
PFS	progression-free survival
RT	radiotherapy
RWD	real-world data
RMST	restricted mean survival time
sCRT	sequential chemoradiotherapy
SoC	standard of care
TDLR	time to death or local relapse
TMB	tumour mutational burden
TME	tumour microenvironment
TTDM	time to death or distant metastasis
UVA	univariate analysis
VMAT	volumetric arc therapy
