# Supplementary material for: Durvalumab Plus High-Dose Radiation in NSCLC III: Long-Term Results from the Austrian Radio-Oncological Lung Cancer Study Association Registry (ALLSTAR)
Source: Cancers (Basel). 2026 Oct 1;18(19):3173. doi: 10.3390/cancers18193173 (PMC13664182; doi:10.3390/cancers18193173)

Supplementary Figure S1: Impact of performance score (ECOG) on overall survival (OS). (a) Patients with a better performance score (ECOG 0-1; n=211; events: 85) had significantly better OS (mOS = 50.6 months; 95%-CI: 39.4-not reached) compared to those with a higher ECOG of 2-3 (n=17; events: 12; mOS = 18.4 months; 95%-CI: 14-not reached): HR = 3.1; 95%-CI: 1.6-5.6; log-rank p-value <0.0002 (Schoenfeld residuals test p-value = 0.66). (b) The restricted mean survival time (RMST) analysis at 36 months revealed a difference in favour of better performance score (ECOG 0-1) of 10.2 months (Wald test p-value 0.00016).

a)

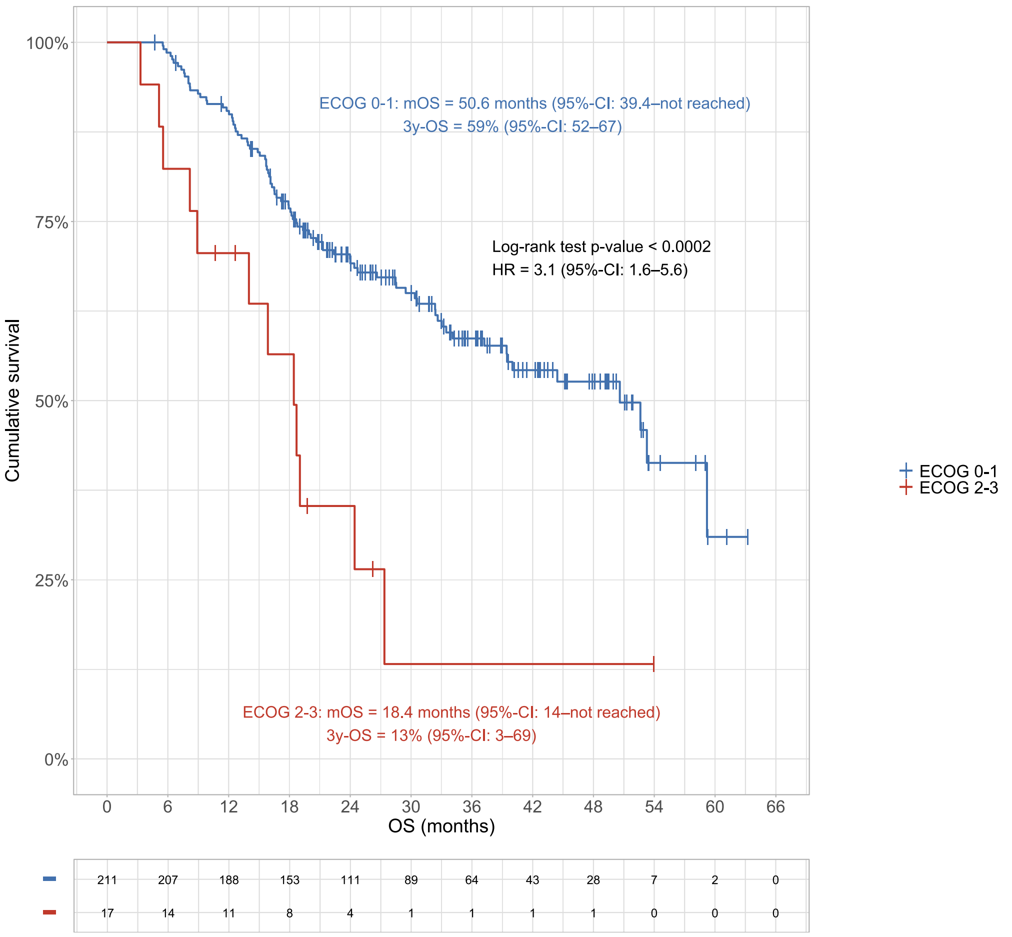

b)

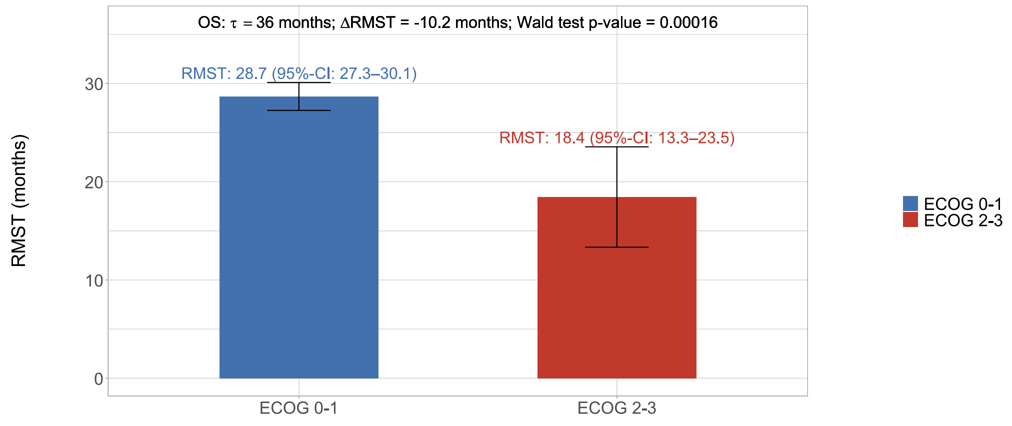

Supplementary Figure S2: Impact of performance score (ECOG) on time to death or metastases (TTDM). (a) Patients with a better performance score (ECOG 0-1; n=211; events: 112) had a significantly better TTDM (mTTDM = 30.7 months; 95%-CI: 22.8-43.6) compared to those with a higher ECOG of 2-3 (n=17; events: 13; mTTDM = 17.8 months; 95%-CI: 8.2-not reached): HR = 2.3; 95%-CI: 1.2-4.1; log-rank p-value <0.004 (Schoenfeld residuals test p-value = 0.68). (b) The restricted mean survival time (RMST) analysis at 36 months revealed a difference in favour of better performance score (ECOG 0-1) of 8.6 months (Wald test p-value 0.0011).

a)

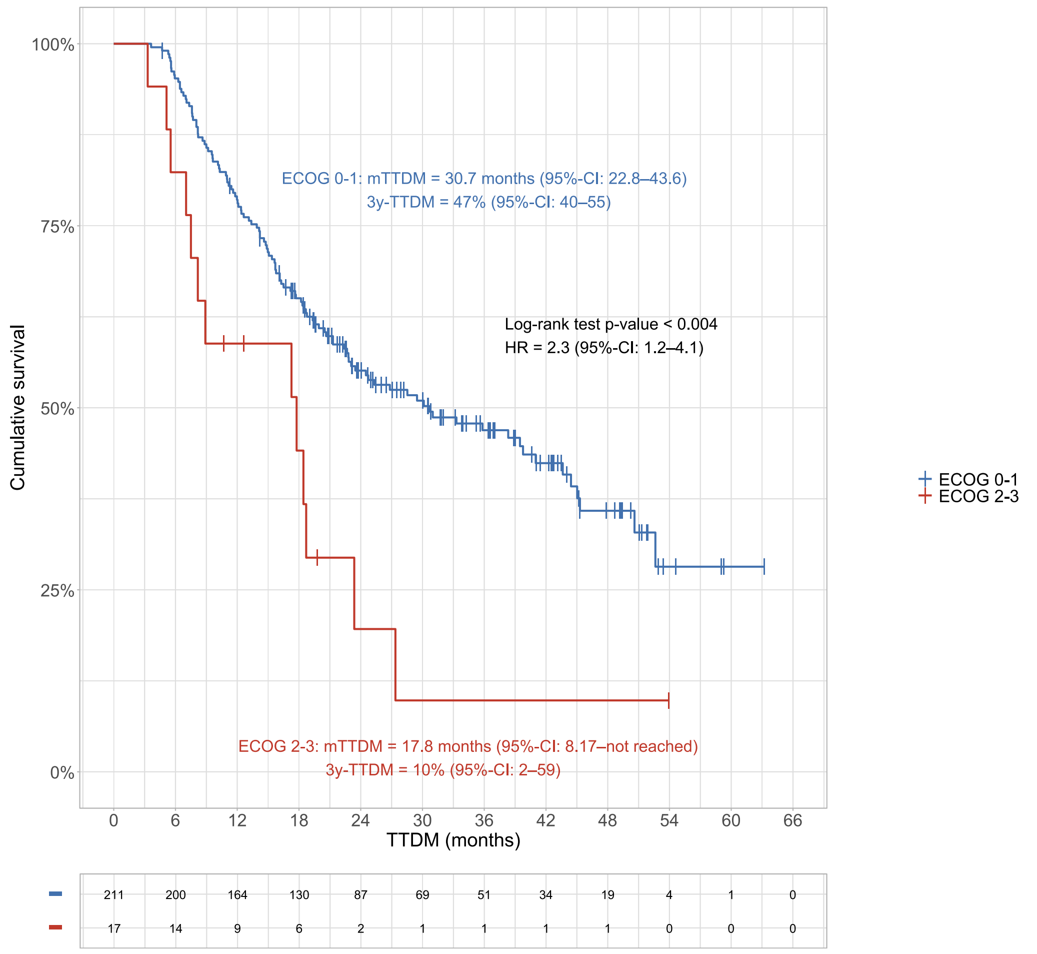

b)

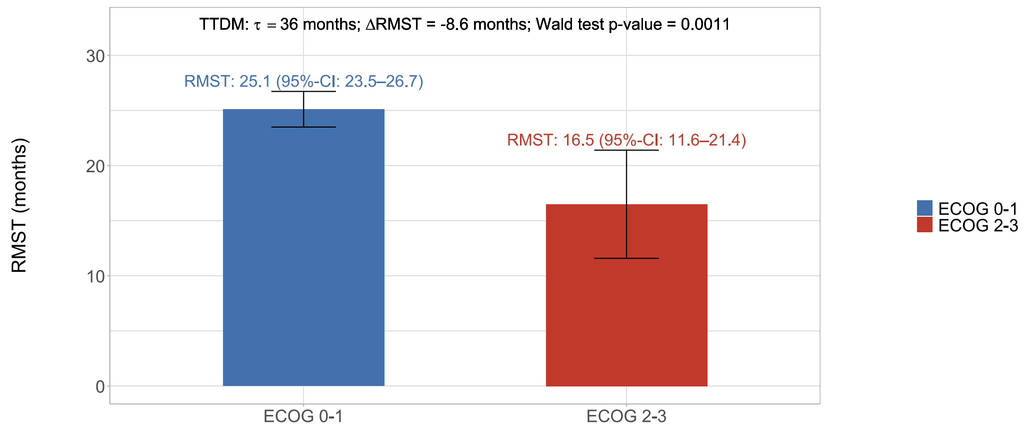

Supplementary Figure S3: Impact of performance score (ECOG) on time to death or local relapse (TDLR). (a) Patients with a better performance score (ECOG 0-1; n=209; events: 113) had a significantly better TDLR (mTDLR = 28.5 months; 95%-CI: 22.7-39.8) compared to those with a higher ECOG of 2-3 (n=17; events: 13; mTDLR = 15.9 months; 95%-CI: 9.0-not reached): HR = 2.5; 95%-CI: 1.4-4.4; log-rank p-value <0.002 (Schoenfeld residuals test p-value = 0.59). (b) The restricted mean survival time (RMST) analysis at 36 months revealed a difference in favour of better performance score (ECOG 0-1) of 9.5 months (Wald test p-value 0.00014).

a)

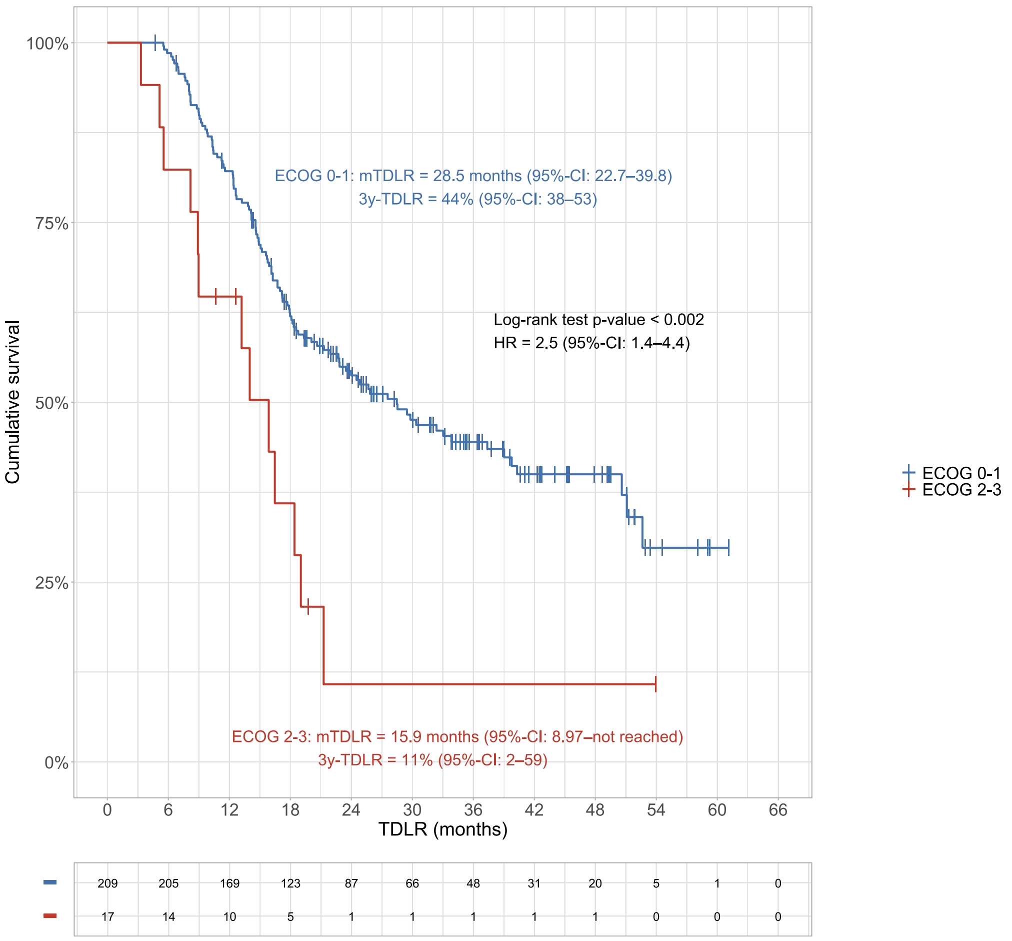

b)

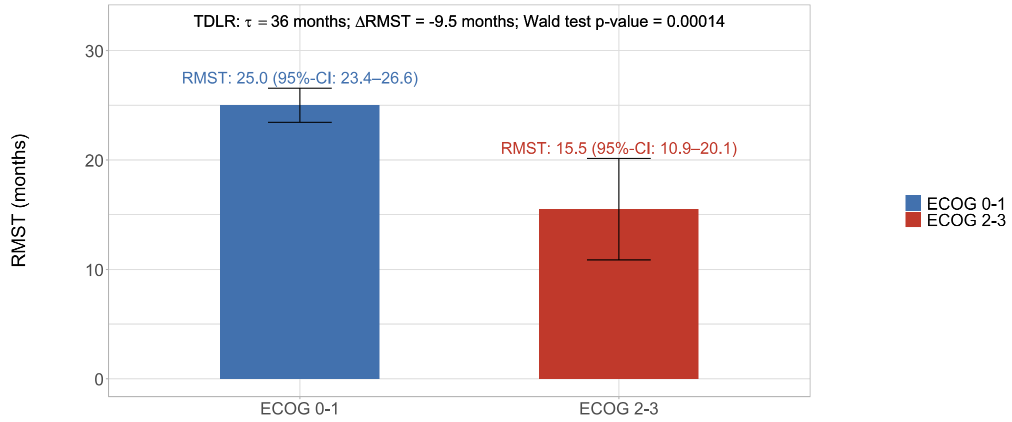

Supplement: Supplementary file 1 [file cancers-18-03173-s001.zip › cancers-4538560-supplementary.pdf]
